# Characterization of VAR2CSA-deficient *Plasmodium falciparum*-infected erythrocytes selected for adhesion to the BeWo placental cell line

**DOI:** 10.1186/1475-2875-7-51

**Published:** 2008-03-26

**Authors:** Francisca Yosaatmadja, Katherine T Andrews, Michael F Duffy, Graham V Brown, James G Beeson, Stephen J Rogerson

**Affiliations:** 1 Department of Medicine, University of Melbourne (RMH/WH), Post Office Royal Melbourne Hospital, Parkville VIC 3050, Australia; 2 Infectious Diseases and Immunology Division, Queensland Institute of Medical Research, QLD, Australia; 3 Australian Centre for International and Tropical Health, a joint program of the QIMR and the School of Population Health, University of Queensland, Brisbane, QLD, Australia; 4 The Nossal Institute for Global Health, University of Melbourne, Parkville VIC, Australia; 5 The Walter and Eliza Hall Institute of Medical Research, Parkville VIC, Australia

## Abstract

**Background:**

Malaria in pregnancy is characterized by accumulation of infected erythrocytes (IE) in the placenta. The key ligand identified as mediating this process is a *Plasmodium falciparum *erythrocyte membrane protein 1 family member, termed VAR2CSA. VAR2CSA appears to be the main ligand responsible for adhesion to chondroitin sulphate A (CSA). Whether other PfEMP1 molecules can also mediate placental adhesion, independent of CSA binding, is unclear.

**Methods:**

The parasite line CS2 carrying a disrupted *var2csa *gene (CS2KO) was selected for adhesion to the BeWo choriocarcinoma cell line, which has been proposed as a model for placental malaria. The selected and control IE were tested for adhesion to placental sections and flow cytometry was used to measure recognition of IE by three serum sets from malaria-exposed men and women.

**Results:**

Wild-type CS2 adhere to BeWo and placental tissue via CSA. CS2KO IE were successfully selected for adhesion to BeWo, and adhered by a CSA-independent mechanism. They bound to immobilized ICAM-1 and CD36. BeWo-selected CS2KO bound at moderate levels to placental sections, but most binding was to placental villi rather than to the syncytiotrophoblast to which IE adherence occurs *in vivo*. This binding was inhibited by a blocking antibody to CD36 but not to ICAM-1. As expected, sera from malaria-exposed adults recognized CS2 IE in a gender and parity dependent manner. In one serum set, there was a similar but less pronounced pattern of antibody binding to selected CS2KO IE, but this was not seen in two others. One *var *gene, It4*var19*, was particularly abundant in the selected line and was detected as full length transcripts in BeWo-selected IE, but not unselected CS2KO.

**Conclusion:**

This study suggests that IE with characteristics similar to the CS2KO have a limited role in the pathogenesis of placental malaria. VAR2CSA appear to be the major ligand for placental adhesion, and could be the basis for a vaccine against pregnancy malaria.

## Background

Almost half of the world's population is exposed to the risk of malaria infection, and despite current control measures, more than one million people die annually. Those who are at the greatest risk are pregnant women and children under five years of age [1,2]. Pregnancy-associated malaria (PAM) is particularly frequent in women in their first and second pregnancies [3]. PAM is characterized by the accumulation of mature stage parasites in the intervillous space of the placenta [4,5]. This sequestration contributes to adverse pregnancy outcomes such as maternal anemia and low birth weight babies [2].

The accumulation of *Plasmodium falciparum*-infected erythrocytes (IE) in the maternal placental intervillous space is thought to be mediated by interactions between members of the *P. falciparum *erythrocyte membrane protein 1 (PfEMP1) family of proteins expressed on the IE surface and placental receptors such as chondroitin sulfate A (CSA) and hyaluronic acid (HA) [6,7]. CSA is expressed by the placental syncytiotrophoblast layer, which lines the maternal blood spaces of the placenta [6,8]. PfEMP1 is also the dominant parasite derived variant surface antigen (VSA) expressed on IE, and immunity to placental malaria has been correlated with development of immunity to a PfEMP1 molecule, termed VAR2CSA (reviewed in [5]).

PfEMP1 is encoded by the *var *gene family. Switching of *var *gene expression alters the PfEMP-1 displayed on the red cell surface and may change the adhesion and antigenic phenotype of the IE, resulting in evasion of splenic clearance [9,10]. The VAR2CSA PfEMP-1 has been linked to the pathogenesis of malaria in pregnancy. Its transcription is associated with adhesion to CSA and HA and with placental malaria [11-14]. VAR2CSA recombinant domains are recognized by IgG from residents in endemic areas, in a gender specific and parity dependent manner [15]. Following genetic disruption of *var2csa *there is little or no binding to CSA [16,17]. It is presently unknown whether VAR2CSA is the only molecule responsible for placental infection. Other *var *genes can be detected in placental malaria [14], and unidentified placental receptors appear to exist [18].

To determine whether other parasite ligands than VAR2CSA mediate placental sequestration, a parasite line with a targeted disruption of *var2csa *(CS2Δvar2csa, referred to as CS2KO) [17] was selected for adhesion to the BeWo human choriocarcinoma cell line [18-20]. CS2KO IE were then characterized for their ability to adhere to human placental tissue and to individual ligands, for the ability of sera from pregnant and non-pregnant adults to recognize the VSA they expressed, and for expression of dominant *var *transcripts.

## Materials and methods

### Plasmodium and human choriocarcinoma (BeWo cells) culture

The *P. falciparum *CS2KO clone [17] and CS2 wild type were cultured as previously described in RPMI-HEPES supplemented with NaHCO3, gentamicin, and 0.5% Albumax II (Gibco) [21]. Parasite line E8B was cultured the same medium, but supplemented with 5% serum and 0.25% Albumax II. To sustain the expression of knobby parasites, trophozoite-stage IE underwent weekly flotation in 0.75% gelatin in RPMI-HEPES [22]. Parasites were maintained in malaria gas mix (5% CO_2_, 1% O_2 _in N_2_).

The human choriocarcinoma-derived cell line BeWo was a gift from Robin Mortimer (Placental Transport Unit, QIMR, Australia). BeWo cells were cultured in RPMI 1640 (Invitrogen)-L-glutamine, supplemented with 10% fetal bovine serum (Invitrogen), and 1% penicillin and streptomycin solution. The cells were grown in a humidified atmosphere in 5% CO_2_.

### Selection of IE for adhesion to BeWo cells

To select CS2KO IE on BeWo cells, trophozoite-stage IE were purified by flotation in 0.5% or 0.75% gelatin in RPMI-HEPES, to 70–80% parasitaemia. The assays were carried out in cytoadherence medium (RPMI-HEPES pH6.8, supplemented with 10% human serum). BeWo cells were grown to ~80% confluence in a tissue culture-treated dish (Falcon, 150 × 22 mm) for up to 48 hr prior the adhesion assays. The cells were washed once with cytoadherence medium before selection. Purified IE were resuspended, and co-incubated on BeWo cells for 45 minutes at 37°C in the malaria gas mixture. Unbound cells were gently washed off with RPMI-HEPES, and bound IE detached by aspiration with parasite culture medium directly onto BeWo cells. Detached IE were then returned to culture with fresh erythrocytes. After each round of selection, IE were grown to ~6–8% parasitaemia, and binding levels were evaluated before reselection. This cycle was repeated ten times on BeWo cells.

### Cytoadherence to BeWo cells

To evaluate adhesion, IE at 1% haematocrit and 6–8% parasitaemia in cytoadherence medium were added to triplicate wells of BeWo cells (~80% confluent) cultured in 6-well plates, with or without 10 μg/ml of bovine trachea CSA (Sigma). After 45 min of co-incubation in malaria gas mix at 37°C, unbound cells were removed by gentle washing with RPMI-HEPES, pH6.8. Bound IE were fixed using 2% glutaraldehyde and stained with Giemsa (Merck). The mean (± SEM) number of adherent IE per 100 BeWo cell nuclei was determined in two independent experiments, by counting under oil immersion (100 × magnification).

To examine the effect of an ICAM-1 blocking antibody (15.2, Serotec) on IE adhesion to BeWo, 8–10 mm diameter wells were created in 6 well plates using a wax pen (Dako) prior to plating. When BeWo cells reached 70–80% confluency they were then co-cultured with IE at 6–8% parasitaemia in cytoadherence medium, with or without anti-ICAM-1 antibody at 10 μg/ml, and experiments performed as described above.

### Adhesion to immobilized receptors

Adhesion to purified receptors was carried out as previously described [21,23]. Purified receptors used were bovine trachea CSA (Sigma), CD36 (R&D Systems), ICAM-1 (Bendermed Systems), human chondroitin sulphate proteoglycan (purified from placental blood; Malaria Research and Reference Reagent Resource Center), HA (from bovine vitreous humour; Sigma) and Fibrinogen (from human plasma; Calbiochem). These receptors were immobilized on plastic Petri dishes (150 mm-diameter, Falcon 1058) as previously described, with some minor adjustments [21]. Briefly, three spots containing selected receptors were blocked with casein (Pierce Biotechnology, Rockford IL) for 30 min at RT, and then washed twice with PBS before adding IE diluted in cytoadherence medium (6–8% parasitaemia and 2% haematocrit). Cultures were incubated at 37°C in a moist box for 45 min, then unbound erythrocytes were removed by gentle washing with RPMI-HEPES. After fixing in 2% glutaraldehyde and staining with Giemsa, the number of adherent IE/mm^2 ^was determined in two to three independent experiments, by counting under oil immersion (100 × magnification).

### Adhesion to frozen placental sections

Adhesion assays on *ex vivo *placental tissue were performed as described previously [24]. Briefly, gelatin-purified trophozoite-stage IEs, that had been grown in RPMI 1640 containing 0.5% Albumax II, were resuspended at 5 × 10^6 ^IE/ml in cytoadherence medium. IE were incubated at room temperature on unfixed sets of three consecutive 5-μm cryosections of normal human placenta. Adhesion was carried out for 1 h, with agitation every 20 min, followed by gentle washing in RPMI 1640 (pH 6.8) to remove unbound IE. CS-specific adhesion was determined by pre-incubating placental tissue with 0.5 units/ml chondroitinase ABC (cABC; Fluka) for 30 min at 37°C, washing three times in RPMI 1640 (pH 6.8), and then adding IE. Specific adhesion to CSA was tested by pre-incubating IE with 100 μg/ml CSA for 15 minutes prior to incubating on placental tissue. Adhesion to CD36 or ICAM-1 was tested by pre-incubating placental sections with 0.5 μg/ml anti-CD36 antibody (FA6/152; Beckman Coulter) or 10 μg/ml anti-ICAM-1 antibody. Following adhesion assays, sections were fixed in 2% glutaraldehyde, stained with Giemsa, and the average number of IE (± SEM) per field (10 fields per well) was determined using a 40 × objective. IE adhesion within the intervillous space, directly to syncytiotrophoblast, or within the foetal villous tissue was determined for at least three independent cryosections (sourced from two different placentas). In each case, binding of IE was compared to IE adhesion on an untreated control section present on the same slide. Placental tissue was obtained with approval from the Mater Human Research Ethics Committee, Brisbane, Australia, after informed written consent.

### Sample collection

Serum and plasma samples were collected from pregnant women and men who lived in Blantyre, Malawi or Madang, Papua New Guinea, with informed consent from donors. Three sets of samples were tested. Cohort-one comprised sera collected in late third trimester of pregnancy from HIV uninfected, malaria infected pregnant women from Queen Elizabeth Central Hospital, Blantyre, collected in 2002–4 [25]. Samples from male patients were from fathers of children admitted to the same hospital with severe malaria [21]. Cohort-two comprised unselected pregnant women and men attending outpatient clinics at Modilon Hospital, Madang, collected in 2001–2 [26]. Cohort-three comprised plasma from pregnant women collected at delivery in Blantyre, Malawi, and samples again from fathers of children admitted to the same hospital with severe malaria, collected in 1997–9 [27]. Negative control sera were collected from Australian blood donors. The collection and use of these samples were approved by the College of Medicine Research Committee, University of Malawi, the Papua New Guinea Medical Research Advisory Committee, and the Clinical Research Ethics Committee, Royal Melbourne Hospital Research Foundation, Victoria, Australia.

### Flow cytometry

Flow cytometry to measure antibodies against VSA expressed by IE was carried out according to our previously described method [27]. Trophozoite stage IE (6–8% parasitaemia, 0.1% haematocrit) were incubated with serum at 1/20 dilution, washed, then incubated with rabbit anti-human IgG (DAKO) and Alexa Fluor 488-conjugated anti-rabbit IgG. Parasite nucleic acid was stained with ethidium bromide (10 μg/ml). Samples were washed three times after each step, and assayed using a BD FACScalibur flow cytometer. The level of specific IgG staining was calculated by subtracting the mean fluorescence intensity (MFI) of uninfected erythrocytes from the MFI of the IE [17]. Non-parametric one way ANOVA statistical analysis was used.

### Quantitative RT PCR and Northern Blot

RNA from synchronized ring stage IE ~4–8 hr post invasion was extracted using Trizol reagent (Invitrogen). [28]. Contaminating DNA was removed by treatment with Turbo DNase (Ambion). cDNA synthesis was performed using Superscript III reverse transcriptase (Invitrogen). PCR was performed to amplify cDNAs using previously-published degenerate nucleotides derived from conserved sequences within *var *gene DBL α [29] and DBL β [30] domains. Primers used were DBLα forward GGinosineGCinosineTG(C/T)GCinosineCC(A/G)T(A/T)(C/T)(A/C)G; DBLα reverse TCTTCinosineG(C/T)CCATTCCTCGAACCA; DBLβ forward (DBL_5.3) G(T/A)(C/G)AACA(T/C)(A/T)T(G/T)TGTAC(A/C)TC; DBLβ reverse (DBL_3.2) AATC(G/T)TTG(A/G/T)GG(A/G)AT(A/G)TA(A/G)TC. PCR products were cloned and 20 clones of each product were sequenced.

Absolute quantitation of the cDNAs of the two *var *genes identified by cloning was performed by Q-RT-PCR using standard curves of plasmids containing *var *gene sequences as previously described [13]. Quantitation of 18S rRNA was used to normalize parasite cDNAs and employed the primers GCTGACTACGTCCCTGCCC and ACAATTCATCATATCTTTCAATCGGTA and standard curves of diluted parasite gDNA. The primers used for Q-RT-PCR of the cloned *var *genes were IT4*var*44 (forward TGCTTCATATTTTCGACCAACGT and reverse CAGCGGCATCGGTTTTG) at 6 μM and IT4*var*19 (forward TGATAAATCTGCAATTTGTAATGCTATG and reverse CCAAGGTCGGCGAAACTATATT) at 2 μM concentrations. In addition, *var2CSA*-specific primers were used as described (forward GGATTGACCACACCGTCTTCTT and reverse TGTCCGCGCCACATATGT) at 2 μM. DNAse-treated RNA of CS2KO9 was used as a negative control for all quantitative RT-PCR runs. cDNAs were normalized against quantitation of 18S RNA.

Northern blots, performed as described previously [13], were hybridized at 55°C with radiolabelled probes derived from IT4*var*19 DBLβ and *var *exon2 sequences. The blots were washed twice at 55°C with 2 × SSC 0.1% SDS (exon 2) or at 60°C with 0.5 × SSC 0.1% SDS prior to exposure to film. Probed blots were stripped with boiling 0.5% SDS and absence of signal confirmed by exposure to film prior to re-probing.

## Results

### Selection for adhesion of IE to BeWo cells

**Figure 1:**
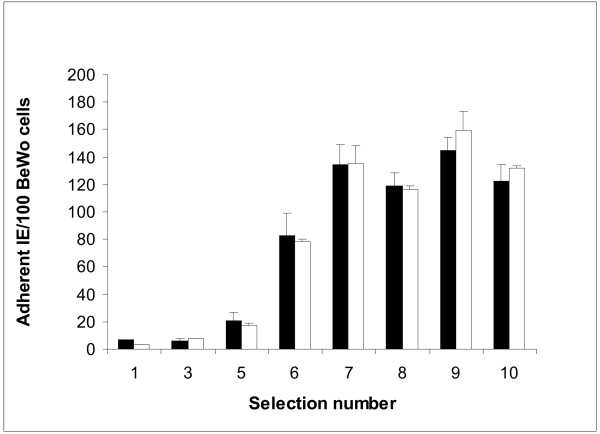
Adhesion of CS2KO IE to BeWo cells following repeated cycles of selection. Adhesion is expressed as IE bound per 100 BeWo nuclei, in the presence (black bars) or absence (white bars) of CSA 10 μg/ml. Data shown are the mean + SEM of at least two independent experiments, performed in triplicate.

CS2 IE bound at high levels to BeWo cells ((343 ± 54 (SEM) IE/100 BeWo) and, as expected [31], binding was inhibited by more than 70% by free CSA (85 ± 36 IE/100 BeWo). By contrast, IE of the *var2csa *knockout CS2 (CS2KO) bound at low levels to BeWo cells (Figure 1), even after five rounds of panning selection. After repeated cycles of selection on BeWo, adhesion of CS2KO increased; after seven cycles adhesion reached a plateau at 120–150 IE per 100 BeWo nuclei (Figure 1). Adhesion was not CSA-dependent, and was around half that of CS2 adhesion to BeWo (145 ± 9; 159 ± 14/100 BeWo nuclei for the selected CS2KO in absence and presence of CSA, respectively). For subsequent characterization CS2KO selection 9 IE (termed CS2KO9) were used.

### Adhesion of CS2KO selected line to immobilized receptors and placental tissue

**Figure 2:**
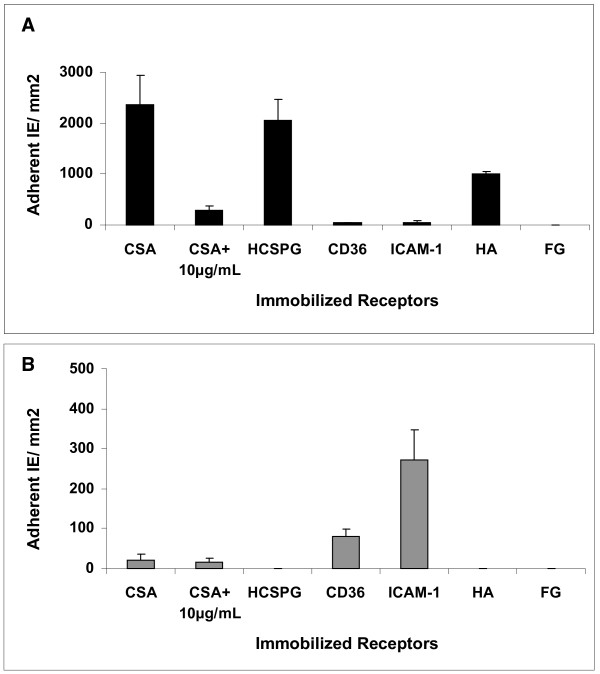
Adhesion of (a) CS2 IE and (b) CS2KO9 IE to immobilized receptors, expressed as IE bound/mm^2^. Data shown are the mean + SEM of two or more independent experiments, performed in triplicate. CSA, chondroitin sulphate A; CSA+10 μg/ml, Adhesion to CSA in presence of free CSA at 10 μg/ml; HCSPG, human chondroitin sulphate proteoglycan; ICAM-1, intercellular adhesion molecule 1. HA, hyaluronic acid; FG, fibrinogen.

The adhesion of CS2 and CS2KO9 IE to purified receptors immobilized on plastic was determined. The adhesion profile of CS2 IE was similar to that previously published (Figure 2a) [7,17,32], whereas CS2KO9 IE adhered to ICAM-1, and to a lesser extent to CD36, with minimal binding to CSA, and none to CSPGs (Figure 2b). Thus, disruption of *var2csa *changes the adhesion profile away from the major placental malaria receptor (CSA), and selection on BeWo does not restore this profile.

To determine whether CS2KO9 IE which adhered to immobilized ICAM-1 also adhered to ICAM-1 on BeWo cells or placental sections, a blocking antibody to ICAM-1 was used. ICAM-1 did not appear to be a major mediator of adhesion to BeWo (CS2KO9, 124.1 (+/- 59.5) IE per 100 BeWo nuclei; CS2KO9 + 10 μg/ml anti-ICAM-1, 125.1 (+/- 47.2) IE per 100 BeWo nuclei). Moreover, the parasite line E8B (which adheres at high levels to soluble ICAM-1) bound to BeWo at low levels (31.0 (+/- 4.0) IE per 100 BeWo nuclei).

**Figure 3:**
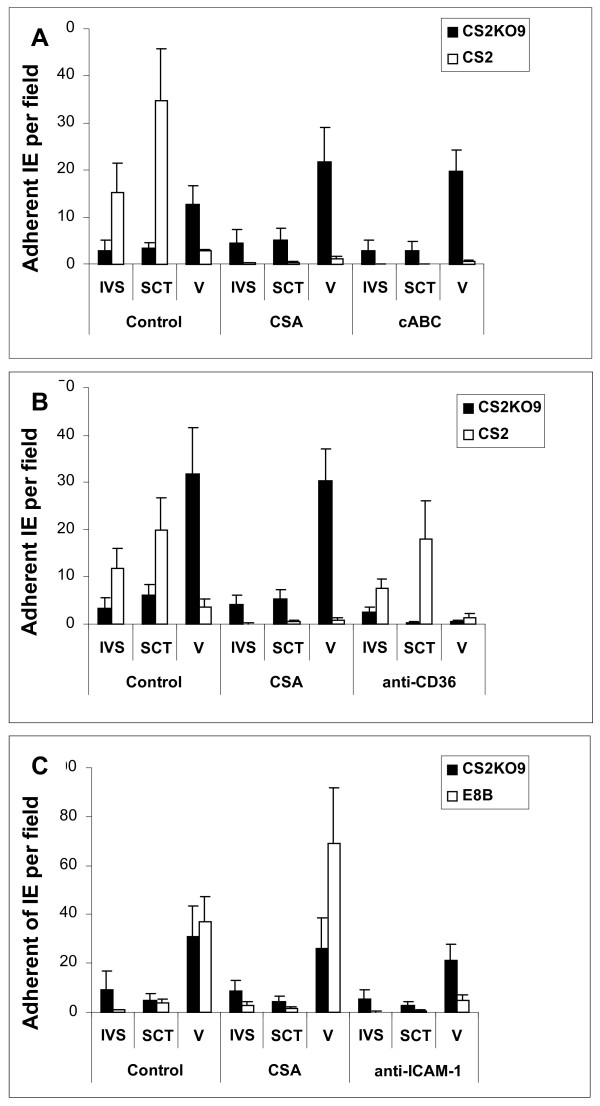
Adhesion to human placental tissue cryosections. Adhesion levels of IE in the intervillous space (IVS), to syncytiotrophoblast (SCT) and over the villus (V) is shown compared to untreated (control) sections present on each slide. (a) Adhesion of CS2 (white bars) and CS2KO9 (black bars) IE in the presence of CSA (100 μg/ml) or following chondroitinase ABC pretreatment (cABC) (0.5 units/mL). (b) Adhesion of CS2 (white) and CS2KO9 (black bars) IE in the presence of CSA or following anti-CD36 pre-treatment (0.5 μg/ml). (c) Adhesion of E8B (white) and CS2KO9 (black) IE in presence of CSA or following pretreatment with anti-ICAM-1 (10 μg/ml). Adhesion was counted at 40× magnification and expressed as average IE bound per field (+SEM) for at least 3 independent experiments.

To evaluate whether CS2KO9 might represent a parasite type able to sequester in the placenta in a CSA-independent manner, adhesion of CS2 and CS2KO9 to *ex vivo *placental tissue sections was investigated [33]. CS2 IE bound to both the intervillous space and the placental syncytiotrophoblast and this binding was inhibited by free CSA and chondroitinase ABC treatment of tissue, consistent with the known distribution of CSA in placenta [8] (Figure 3a). CS2KO9 bound at low levels to both IVS and SCT, and this binding was not inhibited by CSA or chondroitinase ABC (Figure 3a). Adhesion to accessible sites was about 30% of that for CS2 IE, however binding of CS2KO9 IE was predominantly to fetal villous tissue, which is not accessible to IE *in vivo*. This binding may be mediated by CD36, which is known to be expressed in fetal villous tissue [34], as it was significantly inhibited by a blocking antibody to CD36 (Figure 3b). Anti-ICAM-1 did not affect binding of CS2KO9 to placenta (Figure 3c), however binding of a control ICAM-1 binding IE line (E8B), which adheres to the villi and at low levels on the syncytiotrophoblast, was inhibited by anti-ICAM-1 blocking antibody, but not free CSA (Figure 3c).

### Antibodies to CS2KO selected line among pregnant women and men

**Figure 4:**
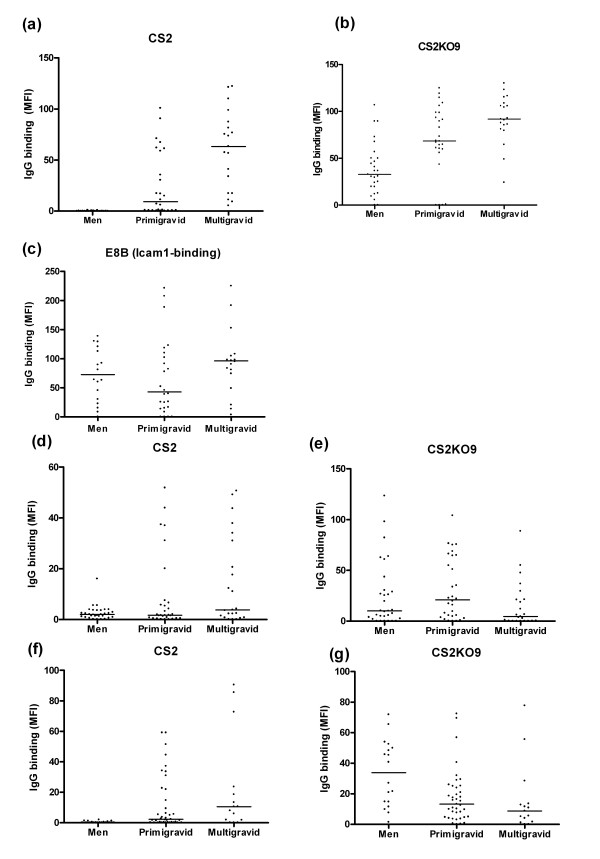
IgG recognition by sera from malaria-exposed individuals. (a) CS2 (b) CS2KO9 and (c) E8B IE were incubated with sera from Malawian men, primigravid and multigravid women (cohort 1). (d) CS2 and (e) CS2KO9 IE were incubated with sera from men, primigravid and multigravid women from Madang, Papua New Guinea (cohort 2). (f) CS2 and (g) CS2KO IE were incubated with sera from men, primigravid, and multigravid women from Malawi (cohort 3). Data presented are mean fluorescence intensities (MFI) for individual samples. Bars indicate median MFI for the population. Significant differences in responses were found as follows: (a) cohort 1, CS2: men and primigravidae (PG; p < 0.0001), men and multigravidae (MG; p < 0.0001) and PG and MG (p = 0.001); (b) cohort 1, CS2KO9: men and PG (p = 0.0011), men and MG (p < 0.0001), and PG and MG (p = 0.047); (c) cohort 1, E8B: men and MG (p = 0.047) (f) cohort 3, CS2, men and PG (p = 0.001), men and MG (p = 0.0013).

Variant surface antigens (VSA) of placental and CSA adherent IE are recognized by sera and plasma from pregnant women in a gender and gravidity-dependent manner. The ability of sera or plasma to recognize CS2 and CS2KO9 IE was assessed, using two cohorts from Malawi and one from Papua New Guinea. For CS2 IE, each cohort showed gender and parity-dependent recognition, although not all differences were statistically significant (Figure 4a,d and 4f). Cohort-one (Figure 4a) showed marked gender and parity dependent recognition of CS2, cohort-three showed significant gender-dependent changes, and cohort-two showed similar trends. A similar, but less pronounced, pattern was seen for CS2KO9 with the first Malawi cohort tested (Figure 4b), but not for E8B which adheres to ICAM-1 and CD36 receptors, but not CSA (Figure 4c), prompting us to test other cohorts. Neither of these showed significant differences by gender or parity in recognition of CS2KO9 (Figure 4e and 4g). We conclude that gender and parity dependent recognition of CS2KO9 occurs uncommonly, and that VSA of CS2KO9 are not prominent targets of pregnancy-specific immunity. The pattern of recognition seen is in keeping with that for other CD36 or ICAM-1 adherent isolates [21].

### *Var *gene expression by CS2KO selected line

**Figure 5:**
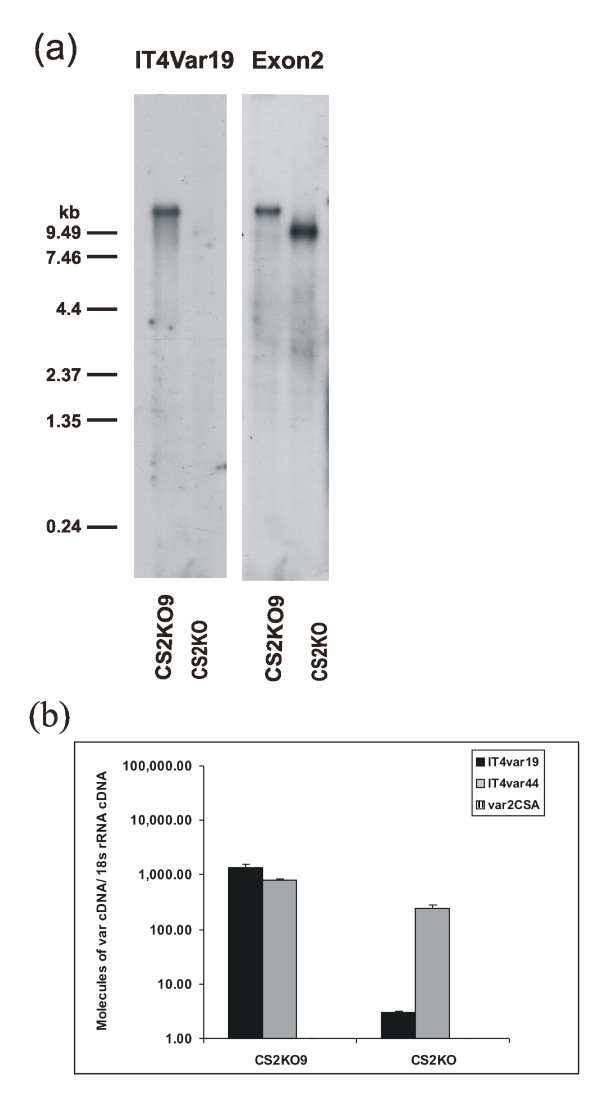
(a) Northern blot analysis of CS2KO9 and CS2KO consecutively probed with IT4*var*19 and *var *exon two sequences (exon2). (b) Quantities of different *var *gene transcripts in BeWo selected CS2KO9 and unselected CS2KO parasites. Absolute quantification of *var *gene transcripts was performed by quantitative RT-PCR using standard curves of plasmids containing the *var *gene target sequences. Presented data is normalized against 18S rRNA levels so that molecules of *var *cDNA from equivalent numbers of cells for each parasite line are compared. Data are presented as mean + standard deviation.

*Var *genes encode PfEMP-1 members, which mediate adhesion to host receptors such as CSA, ICAM-1 and CD36. To investigate whether selection on BeWo cells had resulted in selection for expression of a particular *var *gene, or group of *var *genes, we analysed RNA from CS2KO unselected and CS2KO9 BeWo selected parasites by Northern blot (Fig 5a). A conserved *var *exon 2 probe that hybridizes to most *var *transcripts hybridized to a larger transcript(s) in CS2KO9 than in CS2KO indicating that this larger transcript(s) encoded the BeWo adhesion phenotype. To determine which *var *gene(s) are expressed by CS2KO9, we used RT-PCR amplification of DBLα and DBLβ *var *gene domains to identify transcripts of two *var *genes, It4_*var*19 (accession EF158075) and It4_*var*44 (accession EF158091) (Figure 5) in CS2KO9 parasites. We then used Q-RT-PCR to quantitate levels of transcription of It4_*var*19, It4_*var*44 and *var2csa *in unselected CS2KO, and CS2KO9. Transcripts of *var19 *were much more abundant in CS2KO9 than CS2KO unselected parasites. The dominance of It4_*var*19 in CS2KO9 was confirmed by its co-migration with the most abundant *var *transcripts in CS2KO9, identified by hybridization with the conserved exon 2 probe (Figure 5a). These data indicated that expression of It4_*var*19 contributed to the CS2KO9 phenotype of adhesion to BeWo. Transcripts of It4_*var*44 were only marginally more abundant in CS2KO9 than in CS2KO unselected parasites, indicating that expression of It4_*var*44 was not important for the BeWo adhesion phenotype and, as expected, neither isolate transcribed *var2csa*.

## Discussion

Placental malaria is a distinct form of the disease. Unlike IE infecting non-pregnant individuals, IE isolated from the placenta generally have a highly conserved phenotype (adhesion to CSA), and predominantly transcribe one *var *gene, termed *var2csa *[6,11,12,14,21]. Antibodies that develop naturally in pregnant women cross-react in blocking adhesion of placental isolates from different regions to CSA, suggesting conserved epitopes may mediate protection [35]. *Var2csa *appears to be the most important gene encoding adhesion to CSA and HA; targeted disruption of *var2csa *ablated adhesion to CSA, and after repeated panning little or no CSA adhesion was regained [16,17].

PfEMP1 family members other than VAR2CSA, or different proteins, might also mediate placental adhesion, and IE might accumulate in the placenta by adhering to other ligands expressed on the SCT. To investigate this possibility, CS2KO IE were selected by repeated panning on the BeWo trophoblast cell line, which has been shown to be a useful *in vitro *model of IE adhesion to SCT [18-20,31]. CS2KO IE selected on BeWo gradually increased adhesion to BeWo, which reached a plateau after 7 cycles of panning. Adhesion of selected CS2KO IE remained lower than that of wild type CS2 IE despite repeated cycles of selection, and adhesion was not CSA-dependent. Instead, CS2KO9 IE adhered to ICAM-1, which is known to be expressed on SCT and BeWo cells and upregulated in malaria infection [34]. Importantly, in clinical studies placental IE do not adhere to ICAM-1 [6,21]. Many parasite isolates from children adhere to ICAM-1 [36,37], and adults frequently have antibodies to VSA expressed by such isolates [21], so host immunity may prevent them from establishing infection in pregnant women. ICAM-1 did not appear to be the principal ligand for CS2KO9 IE on BeWo as parasite line E8B, which binds to ICAM-1 and CD36 but not to CSA or HA, adhered to BeWo at significantly lower levels than CS2KO9, and a blocking antibody to ICAM-1 did not significantly decrease adhesion of CS2KO9 to BeWo.

On unfixed snap-frozen placental tissues (Figure 3) CS2KO9 bound at low levels in the intervillous space and to the SCT compared to CS2, and also bound to villous tissue (to which IE have no access *in vivo*), probably using CD36 as a ligand. Adhesion of CS2KO9 was not inhibited by a blocking antibody to ICAM-1. Normal, uninfected placental tissue was used, and it is possible that placental malaria results in induction of expression of other parasite receptors on villi or secreted into the intervillous space. Nevertheless, the present findings suggest that CS2KO IE selected on BeWo bind at low levels to available placental ligands, and that VAR2CSA mediated adhesion is the major driving force behind placental sequestration.

Recently, Viebig et al used the FCR3 parasite line with disrupted *var2csa*[16,17] to study adhesion of FCR3KO parasites to BeWo cells, in experiments similar to those reported here [38]. The overall conclusion was similar, that an intact *var2csa *gene appears essential for IE to show a pregnancy malaria phenotype, but there were some differences in findings. Like CS2KO, the FCR3KO parasites showed some ICAM-1 adhesion; but unlike CS2KO, adhesion to BeWO was partly blocked by anti-ICAM-1. This may be because several *var *genes (at least two abundantly transcribed) were detected in the FCR3KO, whereas by reverse transcription and by Northern blot, we detected a single, different *var *in CS2KO, termed *var19*. FCR3KO parasites selected on BeWo were not recognized in a parity-dependent manner by immune sera, whereas one of our sera sets showed parity dependent recognition of CS2KO, and we saw low levels of adhesion of CS2KO. Neither isolate showed significant specific adhesion to SCT. Together these data suggest that IE panned on BeWo may bind to multiple receptors, but that these are not necessarily relevant to the pathogenesis of placental sequestration or pregnancy malaria.

Gender and parity dependent antibody responses develop in pregnant women, which are directed against VSA expressed by placental or CSA binding IE [39]. These responses have been associated with protection from anaemia, premature birth and low birth weight babies in some studies [40,41]. If IE with a phenotype similar to CS2KO9 are an important cause of placental malaria, then a similar pattern of antibody responses would be expected. By flow cytometry, typical gender and parity dependent responses to CS2 were observed in samples from Malawian and Papuan New Guinean men and women, as expected. CS2KO9 IE were frequently recognized by malaria exposed adults. In one series, similar gender and parity-dependent trends were observed for responses to CS2KO9, but there was no evidence of these in the other series. It is possible that the gender and parity dependent recognition we observed for CS2KO9 reflects the ability of similar variants to elicit pregnancy-specific immunity under certain circumstances. These data suggest that variants expressing similar VSA to It4var19 could be a minor cause of malaria in pregnancy in some cases, but they contribute little to placental sequestration. It4var19 may determine the phenotype of CS2KO9. The receptor for CS2KO9 IE adhesion to BeWo remains unknown.

## Conclusion

The findings presented here support the central role of *var2csa *in placental *P falciparum *infection. When IE lacking *var2csa *were selected for adhesion to the BeWo choriocarcinoma cell line, levels of binding to BeWo and to placental tissue sections were significantly lower than those seen with CS2 IE, which transcribe *var2csa *at high levels, and CS2KO9 IE were not recognized by human sera in a pronounced gender and parity-dependent manner. Variants similar to CS2KO9 frequently circulate in malaria-exposed populations (as demonstrated by frequent presence of antibody in both men and women), but responses to such variants are only occasionally pregnancy-specific. These results further support *var2csa *as a candidate for vaccine development against malaria in pregnancy.

## Authors' contributions

FY cultured BeWo cells and performed selection experiments, flow cytometry and reverse transcription PCR and sequencing and analysed data. KTA examined adhesion to placental cryosections. MFD designed real time primers and analysed results, and performed Northern blots. SJR GVB and JGB conceived the study. SJR FY KTA and MFD drafted the paper, and all authors contributed to, and approved, the final version.

## References

[B1] Greenwood BM, Bojang K, Whitty CJ, Targett GA (2005). Malaria. Lancet.

[B2] Desai M, ter Kuile F, Nosten F, McGready R, Asamoa K, Brabin B, Newman RD (2007). The global burden of malaria in pregnancy: what's known and where are the gaps?. Lancet Infectious Diseases.

[B3] Steketee RW, Nahlen BL, Parise ME, Menendez C (2001). The burden of malaria in pregnancy in malaria-endemic areas. Am J Trop Med Hyg.

[B4] Walter PR, Garin Y, Blot P (1982). Placental pathologic changes in malaria. A histologic and ultrastructural study. Am J Pathol.

[B5] Rogerson SJ, Hviid L, Duffy PE, Leke RF, Taylor DW (2007). Malaria in pregnancy: pathogenesis and immunity. Lancet Infect Dis.

[B6] Fried M, Duffy PE (1996). Adherence of Plasmodium falciparum to chondroitin sulfate A in the human placenta. Science.

[B7] Beeson JG, Rogerson SJ, Cooke BM, Reeder JC, Chai W, Lawson AM, Molyneux ME, Brown GV (2000). Adhesion of Plasmodium falciparum-infected erythrocytes to hyaluronic acid in placental malaria. Nature Medicine.

[B8] Sartelet H, Garraud O, Lorenzato M, Rogier C, Milko-Sartelet I, Huerre M, Gaillard D (1999). Quantitative computer image analysis of chondroitin sulfate A expression in placentas infected with Plasmodium falciparum. J Histochem Cytochem.

[B9] Su XZ, Heatwole VM, Wertheimer SP, Guinet F, Herrfeldt JA, Peterson DS, Ravetch JA, Wellems TE (1995). The large diverse gene family var encodes proteins involved in cytoadherence and antigenic variation of Plasmodium falciparum-infected erythrocytes. Cell.

[B10] Smith JD, Chitnis CE, Craig AG, Roberts DJ, Hudson-Taylor DE, Peterson DS, Pinches R, Newbold CI, Miller LH (1995). Switches in expression of Plasmodium falciparum var genes correlate with changes in antigenic and cytoadherent phenotypes of infected erythrocytes. Cell.

[B11] Salanti A, Staalsoe T, Lavstsen T, Jensen AT, Sowa MP, Arnot DE, Hviid L, Theander TG (2003). Selective upregulation of a single distinctly structured var gene in chondroitin sulphate A-adhering Plasmodium falciparum involved in pregnancy-associated malaria. Mol Microbiol.

[B12] Tuikue Ndam NG, Salanti A, Bertin G, Dahlback M, Fievet N, Turner L, Gaye A, Theander T, Deloron P (2005). High level of var2csa transcription by Plasmodium falciparum isolated from the placenta. Journal of Infectious Diseases.

[B13] Duffy MF, Byrne TJ, Elliott SR, Wilson D, Rogerson SJ, Beeson JG, Noviyanti R, Brown GV (2005). Broad analysis reveals a consistent pattern of var gene transcription in Plasmodium falciparum repeatedly selected for a defined adhesion phenotype. Mol Microbiol.

[B14] Duffy MF, Caragounis A, Noviyanti R, Kyriacou HM, Choong EK, Boysen K, Healer J, Rowe JA, Molyneux ME, Brown GV, Rogerson SJ (2006). Transcribed var genes associated with placental malaria in Malawian women. Infect Immun.

[B15] Salanti A, Dahlback M, Turner L, Nielsen MA, Barfod L, Magistrado P, Jensen AT, Lavstsen T, Ofori MF, Marsh K, Hviid L, Theander TG (2004). Evidence for the Involvement of VAR2CSA in Pregnancy-associated Malaria. J Exp Med.

[B16] Viebig NK, Gamain B, Scheidig C, Lepolard C, Przyborski J, Lanzer M, Gysin J, Scherf A (2005). A single member of the Plasmodium falciparum var multigene family determines cytoadhesion to the placental receptor chondroitin sulphate A. EMBO Rep.

[B17] Duffy MF, Maier AG, Byrne TJ, Marty AJ, Elliott SR, O'Neill M T, Payne PD, Rogerson SJ, Cowman AF, Crabb BS, Brown GV (2006). VAR2CSA is the principal ligand for chondroitin sulfate A in two allogeneic isolates of Plasmodium falciparum. Mol Biochem Parasitol.

[B18] Lucchi NW, Koopman R, Peterson DS, Moore JM (2006). Plasmodium falciparum-infected Red Blood Cells Selected for Binding to Cultured Syncytiotrophoblast Bind to Chondroitin Sulfate A and Induce Tyrosine Phosphorylation in the Syncytiotrophoblast. Placenta.

[B19] Viebig NK, Nunes MC, Scherf A, Gamain B (2006). The human placental derived BeWo cell line: A useful model for selecting Plasmodium falciparum CSA-binding parasites. Exp Parasitol.

[B20] Haase RN, Megnekou R, Lundquist M, Ofori MF, Hviid L, Staalsoe T (2006). Plasmodium falciparum Parasites Expressing Pregnancy-Specific Variant Surface Antigens Adhere Strongly to the Choriocarcinoma Cell Line BeWo. Infect Immun.

[B21] Beeson JG, Brown GV, Molyneux ME, Mhango C, Dzinjalamala F, Rogerson SJ (1999). Plasmodium falciparum isolates from infected pregnant women and children are associated with distinct adhesive and antigenic properties. J Infect Dis.

[B22] Jensen JB (1978). Concentration from continuous culture of erythrocytes infected with trophozoites and schizonts of Plasmodium falciparum. Am J Trop Med Hyg.

[B23] Beeson JG, Brown GV (2004). Plasmodium falciparum-infected erythrocytes demonstrate dual specificity for adhesion to hyaluronic acid and chondroitin sulfate A and have distinct adhesive properties. J Infect Dis.

[B24] Andrews KT, Klatt N, Adams Y, Mischnick P, Schwartz-Albiez R (2005). Inhibition of chondroitin-4-sulfate-specific adhesion of Plasmodium falciparum-infected erythrocytes by sulfated polysaccharides. Infect Immun.

[B25] Mwapasa V, Rogerson SJ, Kwiek JJ, Wilson PE, Milner D, Molyneux ME, Kamwendo DD, Tadesse E, Chaluluka E, Ou CY, Meshnick SR (2006). Maternal syphilis infection is associated with increased risk of mother-to-child transmission of HIV in Malawi. AIDS.

[B26] Beeson JG, Ndungu F, Persson KE, Chesson JM, Kelly GL, Uyoga S, Hallamore SL, Williams TN, Reeder JC, Brown GV, Marsh K (2007). Antibodies among men and children to placental-binding Plasmodium falciparum-infected erythrocytes that express var2csa. Am J Trop Med Hyg.

[B27] Beeson JG, Mann EJ, Elliott SR, Lema VM, Tadesse E, Molyneux ME, Brown GV, Rogerson SJ (2004). Antibodies to variant surface antigens of Plasmodium falciparum-infected erythrocytes and adhesion inhibitory antibodies are associated with placental malaria and have overlapping and distinct targets. J Infect Dis.

[B28] Kyes S, Pinches R, Newbold CI (2000). A simple RNA analysis method shows var and rif multigene family expression patterns in Plasmodium falciparum. Mol Biochem Parasitol.

[B29] Duffy MF, Brown GV, Basuki W, Krejany EO, Noviyanti R, Cowman AF, Reeder JC (2002). Transcription of multiple var genes by individual, trophozoite-stage Plasmodium falciparum cells expressing a chondroitin sulphate A binding phenotype.. Mol Microbiol.

[B30] Kraemer SM, Gupta L, Smith JD (2003). New tools to identify var sequence tags and clone full-length genes using type-specific primers to Duffy binding-like domains. Mol Biochem Parasitol.

[B31] Adams Y, Schwartz-Albiez R, McCarthy JS, Andrews KT (2007). Effect of cytokine treatment on the in vitro expression of the P. falciparum adhesion receptor chondroitin-4-sulphate on the surface of human choriocarcinoma (BeWo) cells. Parasitol Res.

[B32] Rogerson SJ, Chaiyaroj SC, Ng K, Reeder JC, Brown GV (1995). Chondroitin sulfate A is a cell surface receptor for Plasmodium falciparum-infected erythrocytes. J Exp Med.

[B33] Andrews KT, Pirrit LA, Przyborski JM, Sanchez CP, Sterkers Y, Ricken S, Wickert H, Lepolard C, Avril M, Scherf A, Gysin J, Lanzer M (2003). Recovery of adhesion to chondroitin-4-sulphate in Plasmodium falciparum varCSA disruption mutants by antigenically similar PfEMP1 variants. Mol Microbiol.

[B34] Sartelet H, Garraud O, Rogier C, Milko-Sartelet I, Kaboret Y, Michel G, Roussilhon C, Huerre M, Gaillard D (2000). Hyperexpression of ICAM-1 and CD36 in placentas infected with Plasmodium falciparum: a possible role of these molecules in sequestration of infected red blood cells in placentas. Histology.

[B35] Fried M, Nosten F, Brockman A, Brabin BJ, Duffy PE (1998). Maternal antibodies block malaria. Nature.

[B36] Newbold C, Warn P, Black G, Berendt A, Craig A, Snow B, Msobo M, Peshu N, Marsh K (1997). Receptor-specific adhesion and clinical disease in Plasmodium falciparum. Am J Trop Med Hyg.

[B37] Rogerson SJ, Tembenu R, Dobano C, Plitt S, Taylor TE, Molyneux ME (1999). Cytoadherence characteristics of Plasmodium falciparum-infected erythrocytes from Malawian children with severe and uncomplicated malaria. Am J Trop Med Hyg.

[B38] Viebig NK, Levin E, Dechavanne S, Rogerson SJ, Gysin J, Smith JD, Scherf A, Gamain B (2007). Disruption of var2csa gene impairs placental malaria associated adhesion phenotype. PLoS ONE.

[B39] Ricke CH, Staalsoe T, Koram K, Akanmori BD, Riley EM, Theander TG, Hviid L (2000). Plasma antibodies from malaria-exposed pregnant women recognize variant surface antigens on Plasmodium falciparum-infected erythrocytes in a parity-dependent manner and block parasite adhesion to chondroitin sulfate A. J Immunol.

[B40] Duffy PE, Fried M (2003). Antibodies that inhibit Plasmodium falciparum adhesion to chondroitin sulfate A are associated with increased birth weight and the gestational age of newborns. Infect Immun.

[B41] Staalsoe T, Shulman CE, Bulmer JN, Kawuondo K, Marsh K, Hviid L (2004). Variant surface antigen-specific IgG and protection against clinical consequences of pregnancy-associated Plasmodium falciparum malaria. Lancet.

